# n-3 polyunsaturated fatty acids in milk is associate to weight gain and growth in premature infants

**DOI:** 10.1186/1476-511X-8-23

**Published:** 2009-06-26

**Authors:** Sandra M Barboza Tinoco, Rosely Sichieri, Cecília L Setta, Anibal S Moura, Maria G Tavares do Carmo

**Affiliations:** 1 Departamento de Ciências Fisiológicas/Laboratório de Fisiologia Celular da Universidade do Estado do Rio de Janeiro, Rio de Janeiro, Brazil; 2 Instituto de Medicina Social, Universidade do Estado do Rio de Janeiro, Rio de Janeiro, Brazil; 3 Instituto de Nutrição Josué de Castro, Universidade Federal do Rio de Janeiro, Rio de Janeiro, Brazil

## Abstract

**Background:**

Linoleic 18:2 (n-6) and α-linolenic 18:3 (n-3) essential fatty acids and long-chain polyunsaturated fatty acids (LC-PUFA) are essential nutrients for growth and neonatal development. Consumption of preformed n-3 LC-PUFA has been shown to increase gestational duration and to decrease the incidence of premature birth in human studies. This study evaluated the association of essential fatty acids and LC-PUFA in breast milk on the growth of premature children (weight, height and head circumference).

**Study design:**

Thirty-seven premature infants with a gestational age of 37 weeks or less were followed until 6 months of gestational age, adjusted for prematurity. The milk from mothers, weight, height and head circumference measures of children were collected during the follow up. The breast milk fatty acids were quantified by gas-liquid chromatography.

**Results:**

Our results showed that total n-3 PUFA was positively associated with weight gain (*p *= 0.05), height (*p *= 0.04) and body mass index (BMI) of children (*p *= 0.05). Our results also indicate that both linoleic acid and total essential fatty acids were positively associated with BMI and head circumference, whereas oleic acid was positively associated only with head circumference.

**Conclusion:**

These results suggest that the n-3 PUFA composition of milk may be associated with weight gain and growth. Considering the advantages of n-3 LC-PUFA consumption on infant growth and visual function and its association with reduced incidence of premature birth, dietitians should advise pregnant women to increase their intake of foods high in n-3 LC-PUFA.

## Introduction

The n-3 polyunsaturated fatty acids, also known as omega 3 fatty acids, are fatty acids with the first double bound at the third position from the methyl end. They occur in the diet as α-linolenic acid (ALA, C18:3n-3) from vegetable sources and nuts, and as very-long-chain 'marine' n-3 polyunsaturated fatty acid (LC-PUFA) from fish and othern seafood. The main forms of marine n-3 LC-PUFA are eicosapentaenoic acid (EPA, C20:5 n-3) and docosahexaenoic acid (DHA, C22:6 n-3) [1]. Typical intake in Western populations is 1–2 g per day for ALA and 0–0.4 g per day for marine n-3 LC-PUFA [2]. The importance of ALA as a dietetic precursor to EPA and DHA in the human body has also been reported [3]; however, various studies have indicated that the conversion of ALA to functional forms of n-3 LC-PUFA, such as DHA and EPA, is not adequate human beings [2,4,5]. Because the placenta lacks desaturase activity and fetal enzyme activity in uterus is very limited, the fetus depends on placental LC-PUFA transfer [6].

Over the past few decades, n-3 LC-PUFAs have been shown to be essential for neurodevelopment [7-10]. These essential fatty acids, including DHA, α-linolenic acid, and other fatty acids of the n-3 series, are indispensable structural components of cellular membranes and are essential for cerebral and retinal growth, especially in the first two years after birth [11-13]. The deposition of DHA in the retina and in the cerebral cortex occur especially during the third trimester of gestation and in the first six months of extrauterine life; therefore, both the last three months of gestation and first few months after birth are particularly vulnerable to developmental deficits, if DHA is limiting [10,14].

The premature babies, in particular, born during the last trimester of pregnancy have been shown to have low levels of DHA in the blood; this has been correlated with their abnormal eye and brain functions as measured by electroretinogram, cortical visual-evoked potential, and behavioral testing of visual acuity [15,16]

In the other hand, consumption of preformed n-3 LC-PUFA has been shown to increase the duration of gestation and to decrease the incidence of premature birth in both human and animal studies [2,17]. Thus, n-3 deficiency may enhance the risk of premature delivery. Therefore, adequate supplementation of n-3 LC-PUFA becomes essential during the prenatal period and is especially important for pre-mature children [18,19]. The premature baby may be particularly at risk for deficiency of essential long chain fatty acids for the following reasons: 1) neonates and pre-mature children have less adipose tissue, and are therefore more prone to fatty acid deficiencies [13,20]; 2) LC-PUFAs are deposited in considerable quantities during the last trimester of gestation for the growth of the brain and the retina, and premature children are not born with these deposits [3,14]; and 3) the metabolism of the fatty acids in premature infants is still immature. The LC-PUFAS, DHA and AA can be synthesized from ALA and LA, although the activity of conversion is low in humans [2,4,5]. The extent of this conversion of ALA in premature children is not precisely known and is, at best, very limited. [3,6,21]

Although many studies have shown that n-3 LC-PUFAs are beneficial to infant development, the effects of maternal levels of these essential fatty acids on the growth of premature infants are not completely understood. Studies investigating the association of n-3 PUFAs in human milk are scarce. The objective of this study is to investigate associations between human milk levels of n-3 LC-PUFA, including essential fatty acids and long chain polyunsaturated fatty acids, and the growth of premature children.

## Materials and methods

### Process of Selecting the Population

Male and female children born prematurely (gestational age less than 37 weeks) in the Department of Neonatology of the Institute Fernandes Figueira – IFF/FIOCRUZ, Rio de Janeiro, Brazil were selected for this study The study was initiated only after written consent of the parent or guardian, and parents were fully informed of the study goals, methods, risks, and benefits before consent was obtained. Both the project and the consent form were approved by the Ethics Committee for Research in Humans of the Institute Fernandes Figueira/Oswaldo Cruz Foundation (IFF/FIOCRUZ, Rio de Janeiro, Brazil) before the study began and its conduction was in accordance with the Declaration of Helsinki. The children underwent ambulatory monitoring until they reached six months of corrected gestational age for prematurity (age of birth in gestational weeks less than 40 weeks–where the gravidic cycle is complete; full pregnancy).

The gestational age (GA) was determined from the information collected from the mothers or, in cases where the information was not precise, an estimate of the GA was made. This estimate was based on the date of the last menstrual period (LMP), which was either reported by the mother or identified by ultrasound at the 20th week of gestation. In cases where there was a lack of information, we evaluated the GA by using the method of New Ballard [22].

Initially, 215 premature children were selected. Of these, 47 were excluded because they presented a form of pathology (carriers of syndromes or genetic birth defects, alterations in the gastrointestinal tract or the respiratory tract, renal dysfunction, neurological disorders, the presence of congenital infections such as rubella, citomegalia, toxoplasmosis, and herpes, or children of mothers carrying the HIV virus), or because they were receiving artificial milk. In the first month of follow-up, there were 3 deaths and 12 losses due to mothers who never returned for the follow-up of their baby. Of the 153 premature children, 50 had no collection of milk at birth (colostrum) because the mothers had initial difficulties in the collection of milk due to the following reasons: refusal (did not cooperate with the study), had no milk before hospital discharge, were already drawing milk for the milk bank, declined due to pain, or did not schedule a time to meet with the researcher upon their return visit to the hospital during the first few days after delivery. Of the 103 children for whom their mother's milk was collected, 38 had milk collected at baseline only (colostrum), and 65 children had milk collected at baseline (colostrum) as well as in the follow-up period. Thus, the study population includes 65 children who were breastfed for at least 1 month of follow-up. Of these 65 children, 37 were randomly selected for analysis of fatty acids in the maternal milk.

For a type I error of 0.05 and a type II error of 0.20 with a standard deviation of 1 unit and a difference of 1 unit, the required sample size would be 32 children. Therefore, for the estimated number of 37 children, it is possible to observe changes to the standard deviations up to 1.53 units at baseline when the samples are independent.

### Collection of Data

The weight, height, and head circumference were obtained by anthropometry once a month at the outpatient visit. The body weight of premature infants was measured in a calibrated digital balance (Fillizola) when the babies were naked. Their length was measured by placing the premature infants in a horizontal decubitus, where they were measured from the crown to the heel. The head circumference was obtained by using an measuring tape at the supra-orbital ridges and at the largest frontoccipital diameter.

The nursing team collected the breast milk during the monthly outpatient visit. One milliliter of milk was collected from each mother and then deposited in identified ependorffs. The milk samples were frozen at -70°C for later analysis.

### Fatty acid analysis

The fatty acids in the breast milk were evaluated by gas chromatography. The extraction, saponification and lipid methylation were performed in duplicate from 200 μl of milk, according to the method of Lepage and Roy [23], which recommends treatment with 2 ml methanol: toluene 4:1 (v/v) solution and 200 μl of acetyl chloride added under light agitation. The methyl esters were quantified by gas-liquid chromatography using a Perkin Elmer Autosystem XL chromatograph equipped with an ionized flame detector (FID) and a software turbochrom. The fatty acids were separated on a 100 × 0.25 mm × 0.20 μm capillary column SP 2560 (Supelco, USA). The gas chromatographic conditions were the same as those described elsewhere [24] The esters were identified by comparison with their retention time with known standards (Sigma, Supelco). The results were expressed as the percentage of total fatty acids.

### Statistical analysis

The selected premature infants were analyzed for changes in body weight, height and head circumference, and these data were correlated with the concentrations of various fatty acids in milk that could be related to growth. The chromatographic analysis was performed on the milk collected from their mothers shortly after delivery (colostrum), and a second assessment of milk was collected between post-partum days 35 and 42 from the mother of the same child (mature milk).

The databases were constructed using *software *EPI INFO [25]. The follow-up data from the cohort of premature infants were analyzed using the *proc mixed *procedure of the SAS statistical package (Statistic Analysis System), version 8.0 (SAS Institute, Cary, NC, USA) [26,27].

For each one of the outcomes: weight, height, body mass index (BMI) and head circumference, regression models included as independent variables the difference between mature milk – colostrum of fatty acids, time and an interaction factor (differences in fatty acids * time). Therefore, if BMI increased during follow-up and this change was related to fatty acids the variable both time and the interaction factor will be statistically significant.

**Figure 1:**
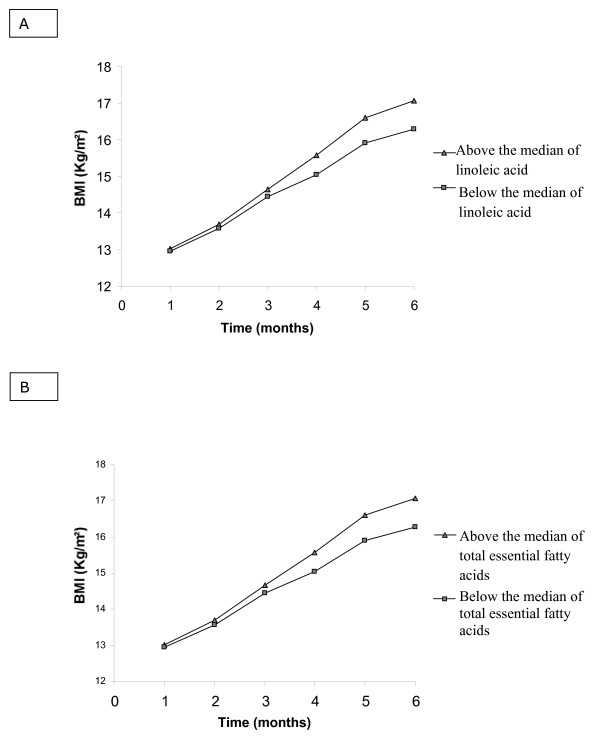
**Estimated values for body mass index (Kg/m^2^) (BMI)**. Model included the difference of linoleic fatty acid (A), and total essential fatty acid (B) between of mature milk and colostrum, time of follow-up (time) and the interaction difference of fatty acids *time. **A – linoleic fatty acid. B **– **total essential fatty acid**.

**Figure 2:**
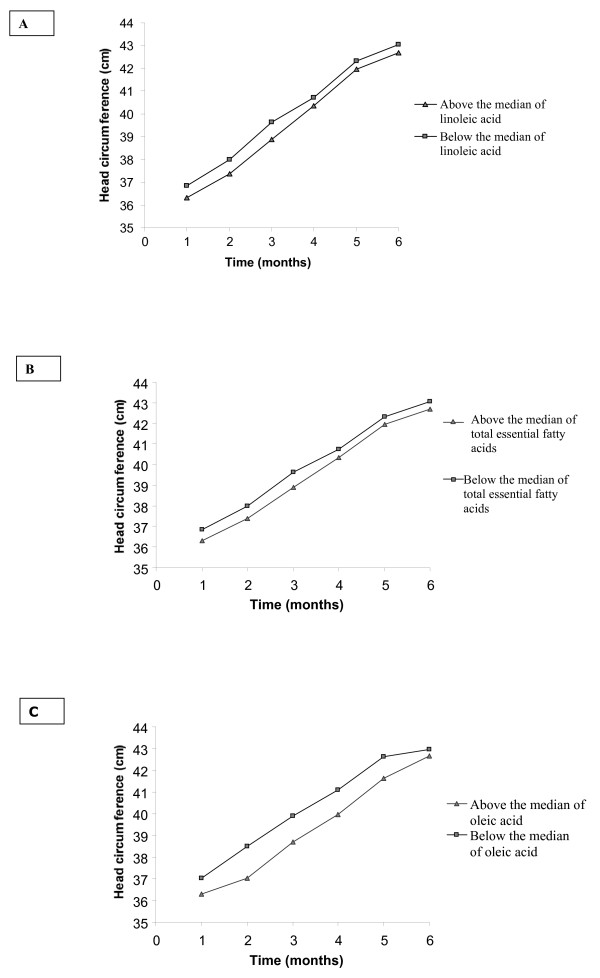
**Estimated values for head circumference (cm)**. Model included the difference of linoleic fatty acid (A), and total essential fatty acid (B), oleic fatty acid (C) between of mature milk and colostrum, time of follow-up (time) and the interaction difference of fatty acids *time. **A – linoleic fatty acid. B – total essential fatty acid. C – oleic fatty acid**.

The difference of values of fatty acids for the two collection times for milk (mature milk and colostrum) was categorized in below the median and above the median allowing plotting two curves for comparisons as in the figures 1 and 2. The difference of values of fatty acids for the two time points is positive when the value for mature milk was higher than that of colostrum, so values above the median also represent increasing levels of fatty acids and vice versa.

## Results

Table 1 shows the anthropometric measures according to the time of follow-up, initiated at birth (0) and up to six months (1 to 6) of life. The nutritional assessment data indicate that female children had an average birth weight of 1790 g and an average height of 43 cm. The average male height and weight at birth were 44.8 cm and 2135 g, respectively. Values of head circumference are also presented in Table 1, but these measures were not registered at birth or during the first month of life. All children exceeded the 2500 g recommended by WHO/OMS at two months of life.

**Table 1:** Number of observations (N), means and standard deviation (SD) of weight, height and head circumference of premature children, by sex and follow-up time.

	***Weight (g)***			***Height (cm)***			***Head circumference (cm)***		
***Time (months)***	***N***	***Mean***	***SD***	***N***	***Mean***	***SD***	***N***	***Mean***	***SD***
Male									
0	21	2135	354	21	44.8	3.80	-	-	-
1	21	3031	1154	21	49.6	4.10	-	-	-
2	19	4434	1286	19	54.3	4.45	17	37.2	2.18
3	19	5566	1457	19	59.6	5.14	16	39.4	2.38
4	15	6449	1715	15	63.3	6.66	15	41.6	2.95
5	12	6545	1232	12	64.0	4.09	10	41.7	1.70
6	11	7183	1407	10	66.5	5.08	10	42.9	1.86
									
Female									
0	16	1790	482	16	43.0	4.32	-	-	-
1	16	2527	542	16	47.1	3.09	-	-	-
2	16	4348	947	16	54.8	4.35	16	37.5	2.12
3	16	5260	966	16	58.9	4.21	15	38.9	2.15
4	16	6040	1056	16	62.2	4.33	15	40.1	1.79
5	15	6694	1149	15	65.1	3.52	13	40.8	1.69
6	9	6945	1153	8	67.1	3.31	7	42.1	1.86

The differences between the fatty acids for the two collection times for milk (mature milk and colostrum) were evaluated in relation to anthropometric variables in the multivariate regression analysis. Gestational age and birth weight, variables known to influence growth, were initially included in the models, but because they did not change the associations of fatty acids and growth they were excluded. Therefore, the models that are presented in Tables 2, 3, 4 and 5 include only the fatty acid differences, time and the interaction between time and the fatty acids.

**Table 2:** Regression coefficient (β) and p-value of the variables included in the regression model for WEIGHT in grams (g)

Fatty acids	Difference of fatty acids		Time* (months)	Difference of fatty acids*time	
	β	*P*	β	β	*P*
**Total *trans*^a^**	- 413.14	0.20	763.02	5.04	0.92
**Total saturated^b^**	138.57	0.67	690.70	45.52	0.37
**C18:1 n-9 (Oleic)**	225.92	0.49	854.56	-65.90	0.19
C18:3 n-3 (α-Linolenic)	565.85	0.07	762.44	-4.50	0.93
C18:2 n-6 (Linoleic)	213.77	0.52	930.34	-114.12	0.02
**Total essentials^c^**	213.77	0.52	930.34	-114.12	0.02
C20:5 n-3 (EPA)	-83.49	0.80	715.28	25.89	0.61
C22:6 n-3 (DHA)	364.97	0.27	944.33	-127.18	0.01
C20:4 n-6 (ARA)	-134.04	0.68	845.94	-62.39	0.22
**Total LC-PUFA^d^**	302.32	0.35	898.21	-101.98	0.04
**Total n-3 PUFA^e^**	615.18	0.05	791.39	-24.87	0.63
**Total n-6 PUFA^f^**	175.32	0.60	860.17	-67.66	0.18

*P-values < 0.0001 for all fatty acids. ^**a **^Includes all isomers of C18:1; ^**b **^includes C14:0, C15:0, C16:0 and C18:0; ^**c **^includes C18:3 n-3 and C18:2 n-6; ^**d **^includes EPA (eicosapentaenoic fatty acid), DHA (docosahexaenoic fatty acid) and ARA (arachidonic fatty acid); ^**e **^includes C18:3 n-3, C20:5 n-3, C22:5 n-3 and C22:6 n-3; ^**f **^includes C18:2 n-6, C18:3 n-6, C20:2 n-6, C20:3 n-6, C20:4 n-6 and C22:4 n-6. PUFA, polyunsaturated fatty acid.

**Table 3:** Regression coefficient (β) and p-value of the variables included in the regression model for HEIGHT in centimeters (cm)

Fatty acids	Difference of fatty acids		Time* (months)	Difference of fatty acids*time	
	β	*P*	β	β	*P*
**Total *trans*^a^**	-1.80	0.10	3.22	0.02	0.92
**Total saturated^b^**	-0.40	0.73	2.84	0.30	0.12
**C18:1 n-9 (Oleic)**	0.88	0.43	3.54	-0.19	0.31
C18:3 n-3 (α-Linolenic)	2.02	0.06	3.33	-0.05	0.78
C18:2 n-6 (Linoleic)	0.98	0.39	3.70	-0.29	0.13
**Total essentials^c^**	0.98	0.40	3.70	-0.29	0.13
C20:5 n-3 (EPA)	-0.14	0.90	2.97	0.19	0.33
C22:6 n-3 (DHA)	1.10	0.33	3.65	-0.27	0.16
C20:4 n-6 (ARA)	-0.75	0.50	3.39	-0.10	0.61
**Total LC-PUFA^d^**	1.15	0.30	3.72	-0.33	0.09
**Total n-3 PUFA^e^**	2.30	0.04	3.48	-0.15	0.43
**Total n-6 PUFA^f^**	0.78	0.49	3.40	-0.10	0.61

*P-values < 0.0001 for all fatty acids. **^a^**Includes all isomers of C18:1; ^**b **^includes C14:0, C15:0, C16:0 and C18:0; ^**c **^includes C18:3 n-3 and C18:2 n-6; ^**d **^includes EPA (eicosapentaenoic fatty acid), DHA (docosahexaenoic fatty acid) and ARA (arachidonic fatty acid); ^**e **^includes C18:3 n-3, C20:5 n-3, C22:5 n-3 and C22:6 n-3; ^**f **^includes C18:2 n-6, C18:3 n-6, C20:2 n-6, C20:3 n-6, C20:4 n-6 and C22:4 n-6. PUFA, polyunsaturated fatty acid.

**Table 4:** Regression coefficient (β) and p-value of the variables included in the regression model for BMI (kg/m2)

Fatty acids	Difference of fatty acids		Time* (months)	Difference of fatty acids*time	
	β	*p*	β	β	*P*
**Total *trans*^a^**	-0.45	0.42	0.68	0.002	0.99
**Total saturated^b^**	0.48	0.38	0.51	0.13	0.27
**C18:1 n-9 (Oleic)**	0.13	0.81	0.81	-0.09	0.45
C18:3 n-3 (α-Linolenic)	0.96	0.08	0.77	-0.05	0.64
C18:2 n-6 (Linoleic)	0.21	0.72	1.12	-0.28	0.02
**Total essentials^c^**	0.21	0.72	1.12	-0.28	0.02
C20:5 n-3 (EPA)	-0.34	0.54	0.54	0.10	0.40
C22:6 n-3 (DHA)	0.76	0.18	1.01	-0.22	0.05
C20:4 n-6 (ARA)	0.16	0.78	0.98	-0.19	0.09
**Total LC-PUFA^d^**	0.80	0.15	1.09	-0.28	0.01
**Total n-3 PUFA^e^**	1.06	0.05	0.82	-0.09	0.43
**Total n-6 PUFA^f^**	0.04	0.94	0.93	-0.16	0.17

*P-values < 0.03 for all fatty acids. ^**a **^Includes all of isomers of C18:1; ^**b **^includes C14:0, C15:0, C16:0 and C18:0; ^**c **^includes C18:3 n-3 and C18:2 n-6; ^**d **^includes EPA (eicosapentaenoic fatty acid), DHA (docosahexaenoic fatty acid) and ARA (arachidonic fatty acid); ^**e **^includes C18:3 n-3, C20:5 n-3, C22:5 n-3 and C22:6 n-3; ^**f **^includes C18:2 n-6, C18:3 n-6, C20:2 n-6, C20:3 n-6, C20:4 n-6 and C22:4 n-6. PUFA, polyunsaturated fatty acid.

**Table 5:** Regression coefficient (β) and p-value of the variables included in the regression model for HEAD CIRCUMFERENCE in centimeters (cm)

Fatty acids	Difference of fatty acids		Time* (months)	Difference of fatty acids*time	
	β	*p*	β	β	*p*
**Total *trans*^a^**	-0.97	0.15	1.22	-0.03	0.74
**Total saturated^b^**	0.26	0.71	0.94	0.16	0.05
**C18:1 n-9 (Oleic)**	0.84	0.23	1.48	-0.21	0.008
C18:3 n-3 (α-Linolenic)	1.11	0.08	0.98	1.14	0.09
C18:2 n-6 (Linoleic)	1.01	0.16	1.60	-0.27	0.0005
**Total essentials^c^**	1.01	0.16	1.60	-0.27	0.0005
C20:5 n-3 (EPA)	-0.22	0.75	0.91	0.17	0.03
C22:6 n-3 (DHA)	0.70	0.31	1.24	-0.04	0.60
C20:4 n-6 (ARA)	-0.48	0.49	1.06	0.07	0.37
**Total LC-PUFA^d^**	0.36	0.61	1.18	-0.006	0.95
**Total n-3 PUFA^e^**	0.92	0.16	0.93	0.17	0.04
**Total n-6 PUFA^f^**	0.61	0.39	1.46	-0.19	0.02

*P-values < 0.0001 for all fatty acids. ^**a **^Includes all of isomers of C18:1; ^**b **^includes C14:0, C15:0, C16:0 and C18:0; ^**c **^includes C18:3 n-3 and C18:2 n-6; ^**d **^includes EPA (eicosapentaenoic fatty acid), DHA (docosahexaenoic fatty acid) and ARA (arachidonic fatty acid); ^**e **^includes C18:3 n-3, C20:5 n-3, C22:5 n-3 and C22:6 n-3; ^**f **^includes C18:2 n-6, C18:3 n-6, C20:2 n-6, C20:3 n-6, C20:4 n-6 and C22:4 n-6. PUFA, polyunsaturated fatty acid.

The regression coefficient *(β) *for weight in grams (g) indicated variations positive over time, which was statistically significant for all fatty acids. The total n-3 PUFA was positively associated with weight gain (*p *= 0.05). The variable of interaction between time and the fatty acid difference estimates the rate of change of weight in relation to different fatty acids, was statistically significant and negative for acid DHA, linoleic, total LC-PUFA, and total essential fatty acids (Table 2).

Table 3 shows estimated values of the coefficients of the fatty acid variables for height in centimeters (cm). Based on these figures, we observed that the total n-3 PUFA was positively associated with a gain in height (*p *= 0.04). The rate of change of growth (difference of fatty acid*time), in relation to all fatty acids was not statistically significant.

Our results showed a positive and statistically significant association (*p *= 0.05) of total n-3-PUFA with BMI. The rate of change of body mass index (BMI) was negative and statistically significant for the DHA, linoleic acid, total LC-PUFA and total essential fatty acids. (Table 4).

The estimated values of the coefficients for head circumference are in Table 5. The rate of change of the head circumference was statistically significant in relation to oleic acid, total saturated fatty acid, EPA, total n-3 PUFA, linoleic acid, total n-6 PUFA and total essential fatty acid. (Table 5).

The estimated values of BMI for values above and bellow the differences in fatty acids based on the regression model which included also time of follow-up and the interaction variable between time and categorized value for the difference of fatty acids are represented in Figures 1 for linoleic acid (Figure 1A), that shows that the smaller reduction in milk of linoleic acid levels was associated with greater increase in BMI. Figure 1B shows the estimate change in BMI according to the variation for total essential fatty acids (α-linolenic and linoleic). The estimated parameters are in Table 4.

Figures 2 shows the estimated values for head circumference, which estimated parameters are in Table 5. We observed that the smaller reduction in milk of linoleic acid levels, the greater the increase in head circumference at follow-up (Figure 2A). In Figures 2B and 2C indicates that there is a greater head circumference increase in children at the follow-up when there is a smaller reduction in the levels of total essential fatty acids (α-linolenic and linoleic) and oleic acid levels in milk.

## Discussion

In this study, we analyzed the effect of fatty acids on anthropometric variables in premature infants, where the results showed that the total amount of n-3 PUFA found in mother's milk was positively associated with weight gain (*p *= 0.05), gain of height (*p *= 0.04) and BMI of premature children (*p *= 0.05) (Table 2, 3 and 4). These data confirm the importance of n-3 PUFA in the development and growth of premature children.

In fact, the n-3 PUFA, including DHA, α-linolenic acid and other fatty acids of the n-3 series, are essential structural components for cell membranes and are important for the growth of the brain and retina, especially in the first two years after birth [12,13]. However, an important aspect to be emphasized in the results of this study was the influence of these n-3 PUFAs not only on weight, but also on height and, consequently, on the BMI in the first months of life of a premature child. This effect can be explained by the fact that n-3 PUFAs are mainly involved in cell growth and multiplication [13], thus exerting a positive effect on weight and height of children. Olsen et al. [28] also demonstrated an association between increased consumption of dietary n-3 PUFA by pregnant women with increasing birth weight of their children. A study involving 8,729 pregnant Danish women found that low fish consumption was a strong risk factor for preterm delivery and low birth weight [29]. Therefore, there is a need for greater maternal intake of n-3 PUFAs during the intra-uterine phase and during the first months of an infant's life [10] because the mother has the fundamental role in the transfer of these fatty acids through the placenta and breast milk [19]. Because n-3 PUFA can influence a wide variety of biological functions, including being essential for the growth and function of the human body, it is important that expectant mothers meet the nutritional recommendations for the consumption these fatty acids in their diets [19].

Because of the inherent problems with assessing diet and maintaining appropriate food composition tables, many investigators are now assessing dietary intake of PUFAs using biomarkers; in general, the concentrations of PUFAs in breast milk reflect the maternal intake of the previous day [30]. Innis [31] found a positive correlation between the content of DHA in milk with visual and language development in breast-fed infants, while a recent Norwegian study found that children of mothers who received supplementation with cod liver oil during pregnancy had an improved mental processing score at four years of age [16]. Assessing the n-3 PUFA levels in the milk of mothers of premature babies, we observed that there was no significant variation in the total amount of n-3 PUFA found in the colostrum compared to mature milk. We hypothesized that the presence of n-3 PUFA in milk may be associated with weight gain and structural development in children.

Although the rate of change in BMI was negative in relation to linoleic acid and total essential fatty acids, our results indicate that both linoleic acid and total essential fatty acid are associated with increased BMI in premature children. In the model adjusted by time of follow-up and the interaction variable between time and categorized value for the difference of fatty acids (difference of fatty acids*time), it can be seen that the smaller reductions in the linoleic acid levels (Figure 1A) and total essential fatty acid (Figure 1B), the greater the increase in BMI during the follow-up period. Similar results were observed for head circumference in relation to linoleic acid, total essential fatty acid and oleic acid (Table 5); the higher the levels of linoleic acid (Figure 2A), oleic acid (Figure 2C) and total essential fatty acids (Figure 2B) in milk, the greater the increase in head circumference during the follow-up. Within the diversity of fatty acids, we can say that not only the LC-PUFAs, but also their metabolic precursors, the essential fatty acids (linoleic and linolenic), are transferred through breast milk to the child and may also exert a positive effect on the health of the child by encouraging early brain development and growth, as was reflected by the increase in BMI. Essential fatty acids also have an important role during the intra-uterine phase, extending the length of gestation and thereby influencing birth weight [3]. The unquestionable necessity of essential fatty acids is demonstrated by these results, so the type of acid that accumulates in fetal tissue can determine weight gain, height gain and increases in head circumference.

In summary, this study demonstrates the influence of maternal lipid consumption during lactation or breastfeeding in the growth of premature infants. It is clear that dietary polyunsaturated fatty acids are transferred into human breast milk and, subsequently, to the child. The results of this study point to the influence of maternal nutrition during pregnancy and lactation on infant growth in the first months of life, reinforcing the need for maternal nutritional education about appropriate consumption of n-3 PUFA and essential fatty acids to support healthy infant growth [9].

In conclusion, we found that the quality of dietary lipids offered through breast milk during the first months of life is a critically important determinant in the of growth premature infants.

## Competing interests

The authors declare that they have no competing interests.

## Authors' contributions

SMB made substantial contributions to conception and design, acquisition of data and also the analysis and interpretation of data. RS participated in the design of the study and performed the statistical analysis and helped to draft the manuscript. CLS participated in the fatty acid analysis. ASM made substantial contributions to conception and design of the study and interpretation of the data and coordination to draft the manuscript. MGTC made substantial contributions to the conception and design, analysis and interpretation of the data and coordination to draft the manuscript. All authors read and approved the final manuscript.
